# Caroli’s disease incidentally discovered in a 16-years-old female: a case report

**DOI:** 10.11604/pamj.2022.41.204.34088

**Published:** 2022-03-14

**Authors:** Abdullatif Almohtadi, Faisal Ahmed, Fawaz Mohammed, Morad Sanhan, Abdulghani Ghabisha, Lina Al-moliki

**Affiliations:** 1 Department of Radiology, School of Medicine, Ibb University of Medical Science, Ibb, Yemen,; 2 Urology Research Center, Al-Thora General Hospital, Department of Urology, School of Medicine, Ibb University of Medical Science, Ibb, Yemen,; 3 Department of Orthopedy, School of Medicine, Ibb University of Medical Science, Ibb, Yemen,; 4 Department of Internal Medicine, Al-Thora General Hospital, School of Medicine, Ibb University of Medical Science, Ibb, Yemen

**Keywords:** Caroli’s disease, abdominal pain, cholangitis, case report

## Abstract

Caroli´s disease is a congenital hepatic disorder characterized by nonobstructive saccular or fusiform dilatation of the intrahepatic bile ducts with the absence of congenital hepatic fibrosis. Caroli´s disease is rare, with few reported cases in the literature, making it hard to distinguish from other liver abnormalities. We present a case of Caroli´s disease discovered indecently in a 16-year-old female who presented with recurrent abdominal pain and intermittent jaundice in the last three years. Abdominal Computed tomography (CT) showed mild liver enlargement with multiple cystic dilatations of the intrahepatic saccular bile ducts cystic dilatations without hepatic fibrosis. The patient was treated conservatively with ursodeoxycholic acid and antibiotic therapy and discharged with regular follow-up. In conclusion, Caroli´s disease should be considered in the differential diagnosis in patients with recurrent abdominal pain and cholangitis without risk factors or relevant history.

## Introduction

Caroli´s disease is a rare liver congenital malformation with a prevalence rate of less than one in one million societies with a male to female ratio of 1: 1.8 [1]. The main features of Caroli´s disease are intrahepatic bile duct dilation with biliary tract involvement in the focal or multifocal direction, while the absence of congenital hepatic fibrosis distinguishes it from Caroli syndrome [2,3]. The typical symptoms of Caroli´s disease are abdominal pain, itching, jaundice, and recurrent cholangitis, which occurred due to liver insufficiency and portal hypertension [3]. It may associate with splenomegaly, abdominal ascites, edema, coagulopathy, and esophageal varices [4]. Caroli´s disease is rare, with few reported cases in the literature, making it hard to distinguish from other liver abnormalities [4]. Hence, we present a 16-year-old female presented with cholangitis and was diagnosed with Caroli´s disease by computed tomography scan. This study aims to add to current knowledge about this sporadic congenital disease.

## Patient and observation

**Patient information:** a 16-years-old female presented with abdominal pain, low-grade fever, and jaundice three days ago. The pain was mild and was located in the right upper quadrant. She had no history of nausea, vomiting, weight loss, changes in bowel habits, or alcohol consumption. The patient mentioned a history of chronic abdominal pain, recurrent jaundice, and repeated hospital admission due to her pain without improvement. The patient was not a smoker and there was no family history of congenital or hereditary diseases.

**Clinical findings:** the vital signs were stable, and the oral temperature was 37.9°C. The abdomen was mildly distended with mild tenderness in the right upper quadrant and mild hepatosplenomegaly.

**Diagnostic assessment:** the white blood cell count: 12×10^3^/ml, hemoglobin: 11 g/dl, platelets: 260×10^3^/ml, blood urea nitrogen: 14 mg/dl, and creatinine: 1.1 mg/dl, total bilirubin: 5 mg/dl, direct bilirubin: 3.5 mg/dl, albumin: 4.3 g/dL, alkaline phosphatase: 305 U/L, aspartate aminotransferase (AST): 24 UI/mL, and alanine aminotransferase (ALT): 13 UI/ml. The viral hepatitis marker was negative. The coagulation tests: prothrombin time (PT), international normalized ratio (INR) and partial thromboplastin time (PTT) were all within the normal range.

**Figure 1:**
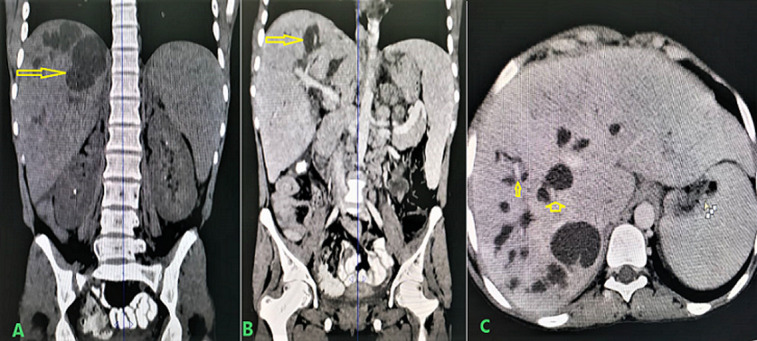
abdominal CT scan showing intrahepatic cystic dilatation (A); non-contract CT scan in coronal views (B); contract CT scan in coronal views (C); contract CT scan in axial views

Abdominal ultrasound (US) showed multiple intrahepatic cysts associated with hepatomegaly and splenomegaly. Computed tomography (CT) scan of the abdomen showed mild enlargement of the liver with multiple multifocal hypodense intrahepatic cystic lesions in all segments and the largest one located at a posterior right upper lope, measuring about 3.6×2.8 cm, and closely connected to intrahepatic biliary ducts without common bile duct dilatation or hepatic fibrosis. Mild portal and liver vein dilatations, hepatosplenomegaly, and mild abdominal ascites were observed (Figure 1).

**Therapeutic interventions:** the patient was newly diagnosed with Caroli´s disease, so conservative medical therapy was started. She has been treated with an intravenous antibiotic; Cefuroxime 750 mg every 8 hours for 5 days, Metronidazole 500mg every 8 hours, Ursodeoxycholic acid every 12 hours and Pantoprazole 40 mg daily.

**Follow-up and outcome:** after five days, the patient's general condition improved, and she was discharged with regular follow-up.

**Patient perspective:** at the 6-month follow-up, the patient reported that she continued taking the prescribed medications. She stated that she felt more energized and could attend school and work more frequently. She also mentioned feeling more rested, which she attributed to the absence of abdominal pain and itching.

**Written informed consent:** written informed consent was obtained from the patient for participation in our study.

## Discussion

The pathophysiology of Caroli´s disease is still unknown. However, a genetic profile seems to be autosomal recessive [4]. An unbalanced translocation between chromosomes 3 and 8 was discovered, implying that loss of distal 3p and/or gain of 8q could be pathogenetic in Caroli´s disease [5]. Possible mechanisms for cystic dilatations in Caroli´s disease are: obstruction of the hepatic artery leads to bile duct ischemia and cystic dilatation, overgrowth of the bile epithelium and connective tissue development, lack of normal molting of the border duct plates with the portal ducts and consequently in the cysts around the portal triads [6,7].

Recurrent abdominal pain in the right upper quadrant area, itching, and jaundice with recurrent bacterial cholangitis are the most manifestations of Caroli´s disease [4]. Our patient was presented with the same manifestations.

The US, abdominal CT, magnetic resonance cholangiopancreatography (MRCP), and magnetic resonance imaging (MRI) are the usual radiologic method for investigations. Despite being operator-sensitive and having low specificity, the US provides the best initial examination because it is noninvasive, simple, and inexpensive. The US findings are unusual dilation of the intrahepatic bile ducts and dilation of the extrahepatic ducts in some cases due to cholelithiasis [4,8]. MRCP is associated with reasonable precision, high predictability, and low potential complications. It is currently the optimal radiographic option. Nevertheless, it is more expensive and used only for determining the disease's nature and severity [6,9]. Abdominal CT scans with intravenous contrast enhancement may reveal central dots in this disease [4,10]. Our patient incidentally detected dilated hepatic cystic lesions on an abdominal CT scan compatible with Caroli´s disease.

There are no exact guidelines for treating Caroli´s disease due to its rarity. However, the proposed therapy should adapt to the clinical manifestations and the potential consequences of biliary abnormalities. Ursodeoxycholic acid is the chosen treatment option in a patient with mild symptoms. Ursodeoxycholic acid has three significant mechanisms of action: the first protects cholangiocytes from the cytotoxic activity of hydrophobic bile acids. The second mechanism is the stimulation of hepatobiliary secretion. The last mechanism is hepatocyte protection against bile acid-induced apoptosis [11]. In patients with symptoms of cholecystitis, instead of ursodeoxycholic acid, a proper antibiotic therapy should be started [3]. The main indication for surgical interventions in Caroli´s disease are biliary obstruction, abscess formation, and bile duct stones. Additionally, patients with an advanced disease stage may qualify for a liver transplant [3,4]. Our patient had mild symptoms treated with ursodeoxycholic acid and proper antibiotics for symptoms of cholangitis.

The differential diagnosis of Caroli´s disease is primary sclerosing cholangitis, recurrent pyogenic cholangitis, polycystic liver disease, choledochal cysts, biliary papillomatosis, and the Von Meyenburg complex [12]. The Von Meyenburg complex usually does not cause symptoms or disturbances in liver functions. MRCP is the optimal choice for its diagnosis, characterized by multiple small-size cysts less than 15 mm and does not communicate with the biliary tree [13].

## Conclusion

Caroli´s disease is a rare congenital malformation of the intahepatic bile ducts. Despite its low prevalence, Caroli´s disease should be considered in the differential diagnosis in patients with recurrent abdominal pain and cholangitis without risk factors or relevant history. Radiologic investigations such as the US, abdominal CT scans, and MRCP are helpful to confirm the diagnosis. Ursodeoxycholic acid is the preferred medical treatment. However, surgical intervention may be required in specific circumstances depending on its location, severity of the disease, and comorbid conditions.
